# Navigating the mind: investigating the complex relationship between mind-wandering and daydreaming

**DOI:** 10.1038/s41598-026-59134-1

**Published:** 2026-07-14

**Authors:** Weronika Hryniszak, Eli Somer, Krystian Barzykowski

**Affiliations:** 1 Applied Memory Research Laboratory, Faculty of Philosophy, Institute of Psychology, Jagiellonian University, Ul. Ingardena 6, 30-060 Krakow, Poland; 2 Doctoral School in the Social Sciences, Jagiellonian University, Krakow, Poland; 3 School of Social Work, University of Haifa, Haifa, Israel

**Keywords:** Mind-wandering, Daydreaming, Task-unrelated thoughts, Attention, Consciousness, Cognitive neuroscience, Human behaviour

## Abstract

Mind-wandering and daydreaming are often treated as synonymous in the literature, yet it remains unclear whether they represent the same cognitive phenomenon in everyday experience. Although mind-wandering and daydreaming are used interchangeably, there are reasons to argue that they relate to two different cognitive phenomena. This study aimed to investigate the differences and similarities between mind-wandering and daydreaming. Using an experience sampling method, participants reported episodes of mind-wandering and daydreaming ten times daily at fixed intervals over seven days. We compared these episodes in terms of phenomenological characteristics and their relative frequency of occurrence. The observed differences were consistent across participant-level analyses and episode-level mixed-effects models, supporting the distinction between mind-wandering and daydreaming as related but separable cognitive phenomena.

The stage 1 protocol for this Registered Report was accepted in principle on 22/01/25. The protocol, as accepted by the journal, can be found at: 10.6084/m9.figshare.28945814

## Introduction

Every day, we encounter a wide variety of off-task thoughts. For example, during a tedious lecture, our minds may wander to unrelated topics, such as contemplating where to have dinner, planning for the upcoming weekend, or pondering solutions to work-related problems. As our minds wander freely from one thought to another, we may become deeply engrossed in our mental realm, momentarily forgetting the external world. Despite being lost in this train of thought, students can return to the lecture when the teacher calls their name. Consider a scenario wherein an individual deviates toward contemplation of a distinct topic or theme rather than actively engaging in the lecture. In this instance, the individual constructs a vivid, monothematic, and internally coherent narrative within their mind, which possesses a propensity for captivating attention. Such mental activity may evoke emotional responses and elicit a broad array of accompanying physiological sensations, such as tactile perceptions or goosebumps. This cognitive phenomenon resembles immersion in an imaginary realm, akin to watching a self-directed movie within the confines of one’s consciousness.

Consequently, redirecting one’s attention toward the external environment becomes notably more challenging than mind-wandering. All the examples in the current literature are considered manifestations of mind-wandering, yet one might get the impression that they are fundamentally different. Mind-wandering involves maintaining a direct and smooth connection with the here-and-now that allows for split attention. Daydreaming, in contrast, entails absorbed attention in internal thoughts.

### Mind wandering in current research

Mind-wandering has been a topic of interest for researchers since the first decade of the twenty-first century^1^. It is often defined as thoughts unrelated to the current events or tasks we are engaged in^2^. Smallwood and Schooler^3^ posited that mind-wandering occurs when executive control disengages from the primary task in order to attend to personal goals. Mind-wandering studies investigate thoughts about the past, e.g.^4^, the future, e.g.^5^, or a combination of both^6–8^. Methodological approaches to studying mind-wandering encompass both laboratory-based techniques, such as task-related or task-unrelated paradigms^9^, and naturalistic investigations, including sampling methodologies such as experience sampling methods^10^. In laboratory settings, studies frequently employ vigilance tasks which involve monitoring the emergence of task-irrelevant cognitions during monotonous activities, with participants required to respond to infrequent stimuli. For example, participants may be instructed to detect vertical lines embedded within a sequence of horizontal lines. Vigilance task programs are designed with the aim of simulating conditions conducive to sustained attention on monotonous and unstimulating tasks, thus reflecting the cognitive dynamics associated with mind-wandering^7,11^. By definition, such conditions reflect the concomitants of mind-wandering. The Sustained Attention to Response Task (SART)^12^ is frequently employed as a paradigmatic example. However, it is not a typical vigilance task as attention needs to be sustained. In this task, participants are instructed to respond to all presented digits, excluding the number 3, e.g.^9,13–15^. These methodological approaches are often complemented by two distinct methods: self-caught or probe-caught. In the self-caught method, participants must identify instances when their thoughts deviate from the task, e.g.^16^. In contrast, the probe-caught method involves periodic sampling, typically querying participants about their engagement in task-unrelated cognitions at predetermined intervals. Moreover, both the probe-caught and the self-caught methods find utility in naturalistic approaches such as the experience sampling method because they enable investigation of mind-wandering in everyday life^17^. In these studies, participants document instances of mind-wandering every time they occur (self-caught method, e.g^18^) and respond to online inquiries at specified intervals to examine mind-wandering in everyday life (probe-caught method, e.g.^19^).

Recently, there has been a noticeable tendency to distinguish specific features of mind-wandering processes. So far, attention has been focused on the element of intentionality, which makes it possible to distinguish between studies on involuntary and voluntary mind-wandering. Research on intentionality, for example, focuses on its relationship to motivation^20,21^, academic performance^22^, or clinical psychology^23,24^. This distinction between intentional and unintentional mind-wandering has made it possible to capture the fuller range of cognitive experiences associated with mind-wandering and to draw more precise and accurate conclusions from research^9^. More precisely, some researchers have traditionally assumed that mind-wandering in laboratory settings occurs unintentionally^25–27^. However, recent studies have challenged this notion by revealing that participants’ mind-wandering episodes are often intentional^9^, thus validating and extending previous studies. It is, therefore, worth exploring further cognitive properties that may give rise to different phenomena. In the present study, we propose that attention engagement may be one such dimension of the cognitive processes underlying mind-wandering.

The use of attention has been prevalent in explaining why mind-wandering occurs, but little attention has been directed towards understanding how it happens. Existing theories elucidating the onset of mind-wandering underscore the significance of attention and executive functions, particularly in constantly refreshing information within working memory. According to the executive function theory of mind-wandering, working memory plays a pivotal role in maintaining attention to the ongoing activity, irrespective of whether it involves an external task or internal thought processes. Individuals with weaker working memory tend to experience less mind-wandering as their attentional resources are directed toward the current task rather than sustaining a stream of thought^3^. McVay and Kane^28^ believe mind-wandering does not inherently require executive resources and working memory. The emergence of task-unrelated thoughts signifies a lapse in the cognitive control needed to focus on the ongoing task, particularly under heightened task demands that necessitate executive resources^29^.

Thus, both models highlight the fluctuation of attention, focusing on whether it is directed internally or externally, without considering the absorption of attention in the process. The dynamic framework explains how mind-wandering changes over time, considering the focus on particular topics. This theory characterizes mind-wandering as aimless thinking, where one freely transitions from one thought to another without focusing on any single thought for long. In contrast, such as in rumination or absorption, attention is concentrated on a single topic^30,31^. Considering only the common definition of mind-wandering, which emphasizes the shift of attention from a task to internal thoughts, even rumination could be considered a form of mind-wandering^30^. This phenomenology of the process relates to how an episode is experienced by the subject (for more details, see Moulin et al.^32^) and provides a starting point for exploring the mechanisms underlying different types of mind-wandering episodes or thought processes. Given the examples in the introduction, it may be argued that they represent two distinct forms of task-unrelated thoughts.

### Mind-wandering and daydreaming: are they distinct or alike?

Indeed, a literature review indicates at least two terms for these phenomena: mind-wandering and daydreaming. While these terms are sometimes used interchangeably, many voices in the literature suggest that they are distinct^33–35^. A factor contributing to their separateness may be the varying involvement of attention engagement processes, but mind-wandering is often synonymous with daydreaming^15,36–40^. Despite the widespread interchangeability of these two terms, there are good reasons to believe that mind-wandering and daydreaming constitute two distinguishable phenomena.

Indeed, a new study by Shimoni and Axelrod^41^ was published regarding differences between the terms mind wandering and daydreaming. The authors investigated how these two terms are perceived. In their study, participants were given the same scenarios of a protagonist engaged in self-generated thoughts, termed ‘mind-wandering’ or ‘daydreaming’.

Notably, scenarios differed in (1) task-unrelatedness (e.g., whether a person was engaged in some task while thinking), (2) intentionality (e.g., whether a person engaged in mind wandering/daydreaming with or without intention), and (3) episode duration (e.g., whether the episode was very short or long). The results demonstrated that participants perceive differences between mind-wandering and daydreaming, defining them through the context of the situation. Thoughts related to planning, worrying, or ruminating were perceived as mind-wandering, whereas recalling past events and fantasizing were perceived as daydreaming. Moreover, an essential distinction of daydreaming was that it was perceived as a task in itself (i.e., without another activity), which aligns with research in clinical psychology, where maladaptive daydreaming (MD) has been found to often occur in the evenings^42^, suggesting that there is more time or opportunity at this time of day for such an absorbing activity. By a definition broadly used in the current literature, such episodes would not fall under the mind-wandering term due to lack of engagement in the task. This study may be considered an important first step in highlighting the value of investigating the potential similarities and differences between mind-wandering and daydreaming. However, while the study by Shimoni and Axelrod^41^ focuses on the extent to which participants exclusively understand the terms ‘mind-wandering’ and ‘daydreaming’ and investigates laypeople’s reasons for using each of these terms in relation to specific situations, there are no studies examining differences between mind-wandering and daydreaming in terms of how they are experienced and processed. Thus, in the present study, we propose distinguishing between mind wandering and daydreaming from the perspective of phenomenological properties. More precisely, we plan to investigate these phenomena by focusing on their phenomenological properties as they are experienced in real time by participants. We further discuss this possibility below.

### The present study

To better understand daydreaming’s specific characteristics, insights from research on intrusive and involuntary memories prove valuable. Studies have shown that phenomena initially studied in clinical contexts often have non-maladaptive counterparts in everyday life, making this line of inquiry particularly relevant. While daydreaming is generally normal, in clinical psychology it can manifest as a pathological immersion in fantasy, where individuals become severely disconnected from their external environment. The most extreme form of this behavior is known as maladaptive daydreaming—an immersive and addictive imaginative activity that disrupts functioning across multiple life domains and causes significant distress^43^. MD content typically takes the form of a narrative, complete with plot and characters, and may feature an idealized version of the self or unfold from a specific perspective. This clinical manifestation of daydreaming closely parallels the general process of imagining events, which requires focused attention on specific thoughts or fantasies.

In clinically oriented literature, the terminology regarding mind wandering and daydreaming is consistently used, but—to the authors’ best knowledge—the term ‘mind-wandering’ does not appear prominently. However, a significant number of papers have specifically focused on daydreaming. This asymmetry is surprising because mind-wandering and daydreaming are often used interchangeably in experimentally oriented studies and papers. The main goal of this study is thus to determine the similarities and differences between mind-wandering and daydreaming, particularly in terms of the phenomenological characteristics of these processes and their content, with a focus on attentional engagement.

There are several reasons for studying the differences between mind-wandering and daydreaming. Recent theoretical research suggests that the potential distinctions between mind wandering and daydreaming might be understood in terms of their immersion and dynamic nature^35^. Thus, they may differ in specific cognitive characteristics; for instance, mind-wandering is characterized by low cognitive engagement, while daydreaming is highly engaging. Moreover, we suggest that an important component of daydreaming could be the sense of presence associated with immersion. Clinical literature indicates that the degree of immersion in maladaptive daydreaming is significant, making the dreaming experience highly satisfying^44^. The sense of presence is, therefore, an essential, potentially addictive component that can lead to maladaptive daydreaming. Secondly, the dynamic nature of these two phenomena can differ: mind-wandering appears to be a sequence of thoughts not directed toward a specific goal without focusing too much on one thought (moving from one thought to another)^30,45^, whereas daydreaming takes the form of monothematic thoughts that absorb attention. Irving^31^ described mind-wandering as purposeless, referring to the lack of guidance in such thought processes. This means that thoughts drift aimlessly, without following a clear direction or focus over time. In contrast, purposeful thoughts are driven by a specific goal or topic, with attention remaining fixed on a particular issue or concern, such as in the case of rumination. We believe that in terms of dynamics, daydreaming can be placed on the same continuum as rumination because of its possible monothematic and engaging nature. In this sense, mind-wandering can be metaphorically compared to casually scrolling through brief TikTok clips or absentmindedly scrolling through social media feeds, capturing snippets of content that do not have a specific focus.

On the other hand, daydreaming can be metaphorically compared to immersing oneself in a full-length movie, where the mind follows a coherent storyline and engages deeply with the characters, thus creating a more prolonged and elaborate imaginative experience. However, unlike a passive viewer, a daydreamer may actively participate in this ‘mental cinema’. They can manipulate, rewind, pause, skip, and watch scenes again, often creating the plot by taking on the roles of scriptwriter, director, and sometimes even actor in the unfolding narrative. This active involvement allows for a highly dynamic and personalized imaginative journey. Fourth, mind-wandering is defined as thoughts unrelated to a task. At the same time, daydreaming is often considered a task in itself (i.e., without an accompanying task), although it may not always be inherently task-oriented and may also accompany another task at hand. Finally, in clinical psychology, the term ‘daydreaming’ is commonly used instead of ‘mind-wandering’, thus implying that, on clinical grounds, they are recognized as separate phenomena. Immersion in a world of thought that ‘cuts off’ a person from the outside world can manifest as maladaptive daydreaming in extreme cases, leading to suffering or dysfunction^46^. This suggests that a non-clinical form of daydreaming may also be an absorbing (but not addictive) cognitive activity that has not been systematically studied. A similar analogy can be drawn in research on involuntary autobiographical memory, which was previously predominantly understood in relation to intrusive memories and post-traumatic stress disorders. However, researchers have gradually acknowledged that involuntary memories may also be experienced in a non-clinical context in everyday life. The proposed study aspires to facilitate a similar shift in our understanding of daydreaming; specifically, we hope to demonstrate that people engage in daydreaming on an everyday basis and, importantly, not exclusively in clinical contexts.

## Methods

All procedures and analyses were conducted in strict accordance with the preregistered Stage 1 protocol unless explicitly stated otherwise. To address the above aims, the study employed an online experience sampling method. Following the methodology outlined by Seli and colleagues^47^, we assessed how mind-wandering and daydreaming manifest in daily life using repeated probes. Participants reported their thoughts ten times per day across seven consecutive days. Prompts were delivered at predetermined intervals between 9:00 and 22:00, occurring approximately every 80 min.

Participants were provided with clarifications of the phenomenological terms used in the questions (e.g., sense of presence, narrative nature of thoughts, depth of engagement). These explanations were intended solely to facilitate accurate comprehension of the items and did not involve introducing predefined categories or types of thoughts. Participants were informed only that people may experience different forms of ongoing thought (e.g., thoughts focused on a single topic versus multiple shifting topics), without being told that these forms correspond to distinct constructs or that particular features were expected co-occur. The sole purpose of these explanations was to ensure a shared understanding of the terminology used in the questionnaire. Subsequently, participants recorded their thoughts using an online sampling method. On a scale created for the study, they responded to questions regarding the main features of mind-wandering and daydreaming, as well as inquiries about the characteristics and content of the process. The study investigated the content of thoughts and explored how these thoughts arise (process phenomenology). Thus, it aligns with the innovative nature of mind-wandering studies. This approach highlights previously overlooked features and distinctions between mind-wandering and daydreaming, particularly in terms of their processual nature, such as attention, absorption and dynamism.

In the present study, we investigated the idea that mind-wandering differs from daydreaming in several ways, such as attentional engagement, sense of presence, dynamics, and narrative nature. More precisely, episodes of thinking characterized by moving from one thought to another without significant engagement will be classified as mind-wandering, whereas those that absorb attention and are deemed to revolve around a singular topic will be categorized as daydreaming. We expected to observe several differences between mind-wandering (characterized by task-unrelated/thoughts flowing aimlessly without much attention paid to them) and daydreaming (the highly attentionally engaging imaginative contemplation of events directed towards a specific objective with a sense of being there). Specifically, we expected mind-wandering to be characterized by dynamic movement from one thought to another (ratings on the scale will be high) and low attentional engagement, sense of presence, and narrativity (ratings on the scale will be low). Daydreaming should exhibit higher ratings for attentional engagement, sense of presence, narrativity, and a dynamic tendency to remain fixed on a single theme. We do not have firm predictions regarding intentionality, temporality, and valence.

### Ethics information

The study was reviewed by the Institutional Research Ethics Committee (approval number: 221.0042.58_2024). The written consent for participation, containing information about anonymity and the possibility to withdraw from the study at any stage without any consequences, was obtained prior to data collection.

### Sampling plan

No data are available regarding the effect sizes of differences between mind-wandering and daydreaming. Therefore, the analysis is based on typical effect sizes (*Cohen’s d*) considered small, medium, and high: 0.2 (η2 equivalent 0.01), 0.5 (η^2^ equivalent 0.06), and 0.8 (η^2^ equivalent 0.14), respectively^48,49^. The required sample sizes for 80% power in the two-tailed test are 199, 34, and 15, respectively. A sample size of about 70 participants was assumed sufficient to detect a medium effect. Sensitivity analysis suggests that this sample size enables the detection of an effect size of 0.34 (η^2^ equivalent 0.03; which may generally be considered closer to a small effect size).

### Participants

A total of 70 participants were recruited (40 women), aged *M* = 28.8 years (*SD* = 8.58, range from 19 to 56). Participants selected for the study were at least 18 years of age. Participants were recruited through advertisements on various media channels, including traditional outlets and online platforms. Participants received a modest reimbursement of 200 PLN for their participation (c.a. 55 USD).

### Materials

To observe mind-wandering and daydreaming under the natural circumstances of their occurrence, an experience sampling method was used to provide information on (1) the circumstances under which these episodes occur (e.g., whether only in situations in which an individual was engaged in a task, or as a task in itself, such as before falling asleep or while relaxing in the bathtub); (2) the content of the episodes; (3) how the process was experienced by the subject right after the episode ended (e.g., the subject promptly answers questions about it); and (4) the frequency of experiencing absorption of attention by thoughts (daydreaming).

**Fig. 1:**
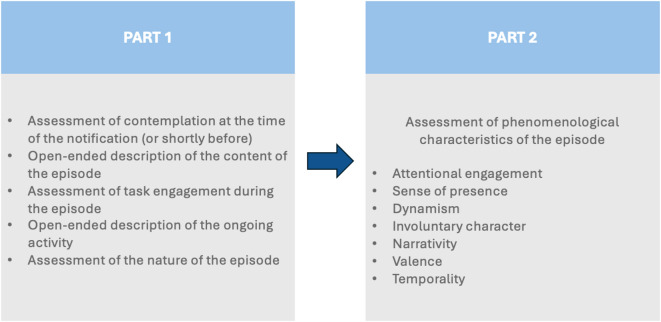
Overview of the procedure. The figure provides a schematic overview of the two parts of the study, illustrating the sequence of assessments and the types of questions participants were asked.

The study was structured into two distinct parts (a detailed overview of the two parts is provided in Fig. 1). In the initial part of the study, participants responded to questions assessing whether they were experiencing an episode of contemplation, which was explicitly defined as a cognitive state distinct from the immediate context (the ‘here-and-now’) and the ‘task-at-hand’. If participants endorsed such instances, they were prompted to briefly describe their ongoing thoughts (e.g., *dwelling on a favorite team’s next game*). Subsequently, participants indicated whether they were engaged in any specific task at the time of the cognitive diversion. They then categorized the nature of the episode in terms of dynamism and attentional engagement by selecting one of three options: (1) contemplating various unrelated subjects with minimal engagement; (2) focusing on a singular topic, with thoughts forming a coherent narrative and sustained engagement; or (3) experiencing thoughts of a different nature. In the second part of the study (see Table 1 for details), and following the methodology utilized in previous studies^50^, participants responded to a series of questions assessing both the content and the processual characteristics of mind-wandering and daydreaming episodes. Using the scale specifically developed for this study, participants rated questions covering seven dimensions: (1) attentional engagement, (2) sense of presence, (3) dynamism, (4) involuntary nature, (5) valence, (6) narrativity, and (7) temporality. Responses were provided on a 7-point scale, with higher values indicating greater intensity (1 = low intensity; 7 = very high intensity). Finally, on the last day of the study, participants were asked whether they consider mind-wandering and daydreaming to be synonymous or distinct phenomena. Detailed instructions and example questions are provided in Table 1.

**Table 1:** Characteristics of mind-wandering and daydreaming: sample questions used in the second part of the probes.

	Instruction	Sample question
Attentional engagement	The following questions ask about how much you were lost in your thoughts Sometimes, you don’t notice what’s happening around you because you’re so wrapped up in your thoughts. For example, you might be thinking and not even hear your coworker calling your name. But other times, you might still be aware of what’s happening while contemplating. For example, you’re lost in thought but still aware of a nearby conversation	Rate the extent to which you were focused on your thoughts during this episode of contemplation: 1—*I was not at all focused on my thoughts* 2—*I was focused to a small extent* 3—*I was focused to a relatively small extent* 4—*I was focused to a moderate extent* 5—*I was focused to a rather significant extent* 6—*I was focused to a significant extent* 7—*I was highly focused on my thoughts*
Sense of presence	Sometimes, you may think about something so vividly that you have a sense of ‘being there’ in your thoughts as if you were deeply involved in those thoughts	Rate how much you were involved in your contemplation: 1—*Not at all involved* 2—*Slightly involved* 3—*Somewhat involved* 4—*Moderately involved* 5—*Quite involved* 6—*Very involved* 7—*Totally involved*
Dynamism	The next question asks about how dynamic your thought flow was and to what degree your thoughts were flowing Sometimes, when your thoughts drift, you’re flowing from one thought to the next in a kind of slide show. For example, in just a short span (could be long or short), you may start thinking about what you’re going to have for dinner, then shift to picturing your upcoming trip to the mountains, and then imagine how you’re going to decorate your house for the holidays. It’s like you’re just moving from one thought to another without skipping a beat However, when your thoughts drift, they may stick to one topic. You might be reviewing your grocery list and thinking about what meal you want to cook with those ingredients. You may even picture yourself walking down the aisles in the store, thinking about where those items are located. Indicate the degree to which all these thoughts are about one thing	Rate the extent your thoughts focused on a single theme or topic: 1—*My thoughts jumped between completely unrelated topics* 2—*My thoughts occasionally related to a single theme but mostly jumped around* 3—*My thoughts sometimes stayed on a single theme for a while but also diverged* 4—*My thoughts moderately focused on a single overarching theme or topic* 5—*My thoughts predominantly centered around one central theme or topic* 6—*My thoughts were highly focused and concentrated on a single theme* 7—*My thoughts exclusively revolved around one theme or topic throughout*
Involuntary character	The next question will address the involuntary nature of mental activity. You may have intended to start the episode, but it could have occurred involuntarily, regardless of your intention	Please indicate how this episode of contemplation came to mind: 1—*The thought simply popped into my mind spontaneously* 2—*I deliberately chose to think about this*
Valence	–	Rate the extent to which the contemplation you described was pleasant: 1—*Extremely unpleasant* 2—*Moderately unpleasant* 3—*Slightly unpleasant* 4—*Neutral (neither pleasant nor unpleasant)* 5—*Slightly pleasant* 6—*Moderately pleasant* 7—*Extremely pleasant*
Narrativity	Please indicate, on a scale from 1 to 7, the extent to which you agree with the following statements about the contemplation episode you just wrote down	My contemplation involved a narrative story in which I was the main character: 1—*Strongly disagree* 2—*Disagree* 3—*Somewhat disagree* 4—*Neither agree or disagree* 5—*Somewhat agree* 6—*Agree* 7—*Strongly agree*
Temporality	–	Please indicate whether your contemplation involved something related to: 1—*Something about the past* 2—*Something about the present* 3—*Something about the future* 4—*Something fantastic and/or fanciful* 5—*Something else* 6—*Two or more of the above temporal elements*

The table presents a comprehensive overview of the categories of questions designed to assess the characteristics of mind-wandering and daydreaming, including dimensions such as attentional engagement, sense of presence, dynamism, involuntary nature, valence, narrativity, and temporality. Additionally, for each category, sample questions are provided.

## Design

A within-subject design was used, with participants reporting their everyday thought activity and identifying instances of mental activity as instances of either mind-wandering or daydreaming.

### Procedure

The study extended over seven days, during which participants received ten daily notifications at fixed pseudo-random intervals asking them to record instances of mind-wandering and daydreaming. We employed dichotomous sampling asking participants to report their current mental state as either focused on an ongoing task or contemplation (e.g., *being lost in thought*) episode, a methodology commonly utilized in studies on mind-wandering^47,51,52^. These notifications prompted participants via SMS at designated times, providing a link to the study hosted on the Qualtrics platform. Participants were encouraged to engage with as many probes as possible.

Participants received detailed instructions outlining the procedures of the study, which they reviewed before initiating their involvement in the study. They were informed of the accessibility of these instructions throughout the entire duration of the study. Participants were also informed that the study focused on exploring the concept of contemplation. The study began with introductory information:

“*Mental thought activity can manifest in various forms. You may experience intense mental immersion, vividly envisioning specific scenarios as if observing a cinematic imagery within your mind. For instance, you might intensely contemplate the intricate details of an upcoming vacation. Alternatively, cognitive absorption may involve a free-flowing stream of thoughts, transitioning seamlessly between different topics without a defined objective. This could entail contemplating plans for tomorrow’s dinner, shifting to considerations of your child’s activities, and concluding with plans for a future summer vacation destination. In this study, we are interested in mental absorption in all its forms, regardless of their nature or content (i.e., what they are, what they are about, and how they manifest).*”

Additionally, on the seventh (final) day of the study, participants were asked an open-ended question regarding their perspective on whether they perceive mind-wandering and daydreaming as synonymous or distinct constructs; they were asked to provide the reasoning behind their viewpoint. This item was included as part of the exploratory component of the study; however, the responses are not analysed or discussed in the present report.

### Results

All analyses were conducted in accordance with the preregistered Stage 1 analysis plan, except where explicitly noted otherwise.

#### Data exclusion and final sample based on probe responses

Participants with incomplete responses were excluded from the analysis. Specifically, data were removed if a participant had responded to fewer than 60% of the total probes. In practical terms, this meant that participants who completed fewer than 42 probes were not included in the final dataset. Because the study aimed to include a minimum of 70 participants, any shortfall resulting from these exclusions was compensated for by recruiting replacement participants. In total, 75 individuals took part, with five additional participants replacing those who did not meet the response-rate criterion. The final sample consisted of 70 participants, who completed on average 53.9 probes (*SD* = 8.73).

#### Descriptive characteristics of episodes

**Fig. 2:**
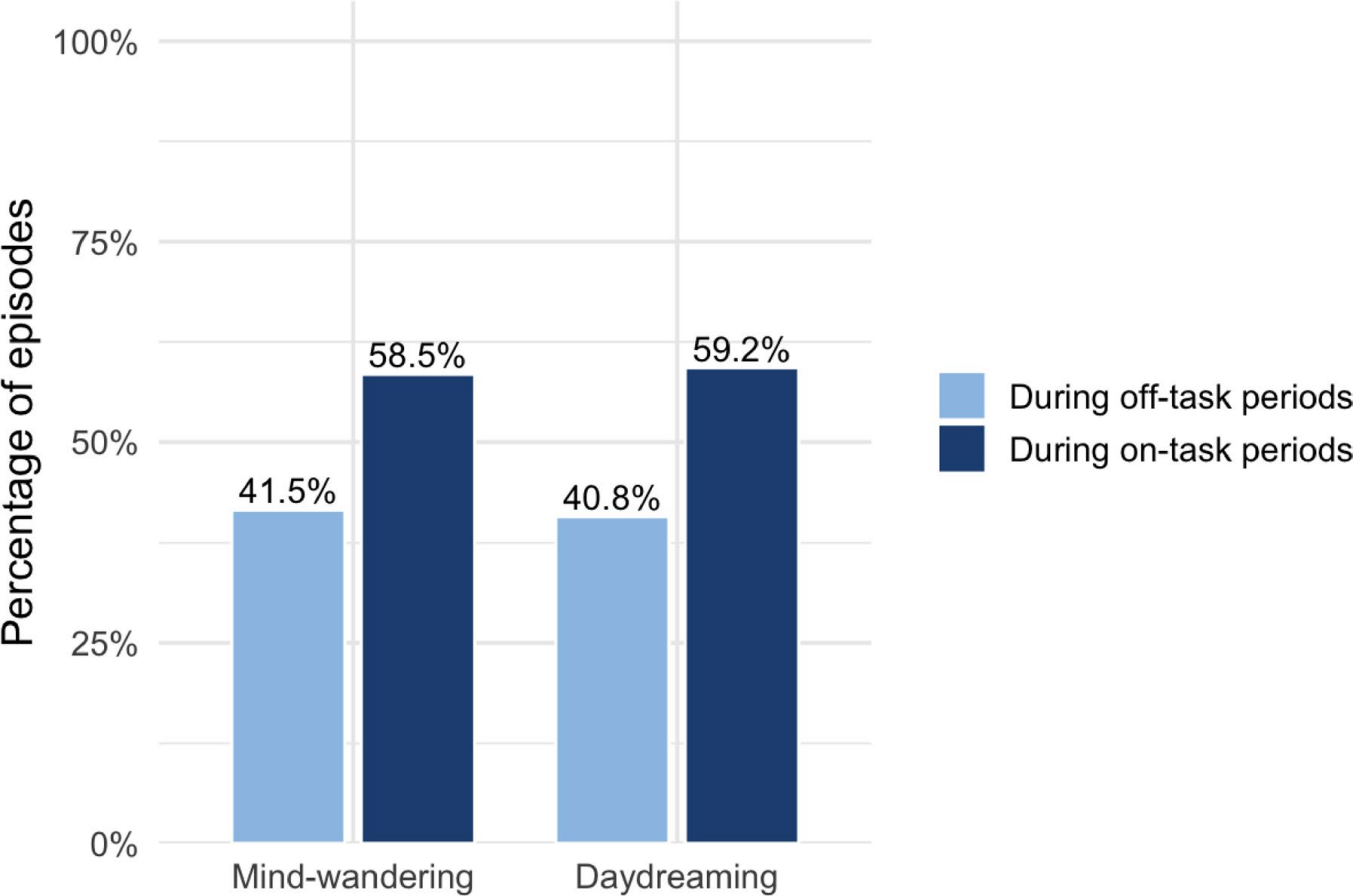
Distribution of mind-wandering and daydreaming episodes by context. The figure shows the proportion of mind-wandering and daydreaming episodes occurring during on-task and off-task periods.

While answering the questions, participants categorized their mental activity as (1) thinking about diverse, unrelated subjects with minimal engagement; (2) focusing on a singular topic, with thoughts forming a coherent story and sustained engagement; (3) focusing on something of a completely different nature. Responses classified as (1) were treated as mind-wandering (MW), while responses classified as (2) were treated as daydreaming (DD). Episodes classified as (3) were excluded from further analysis. Across all participants, 455 MW episodes (*M* = 6.5, *SD* = 6.02, per participant, range: 0–27) and 1190 DD episodes were recorded (*M* = 17.0, *SD* = 11.7, per participant, range: 1–51). To analyse contextual patterns, we examined how often MW and DD occurred during on-task versus off-task periods (see Fig. 2).

Both mind-wandering and daydreaming occurred during task engagement as well as during task-unrelated activity. Although both forms were more frequent during on-task periods (mind-wandering: 58.5%, 266/455; daydreaming: 59.2%, 705/1190), a multilevel logistic regression model indicated no reliable difference in the likelihood of occurring during on-task periods between mind-wandering and daydreaming (OR = 1.12, 95% CI [0.88, 1.43], *p* = 0.77).

#### Phenomenological properties of mind-wandering and daydreaming episodes

##### Participant-level analyses

Participants provided ratings on seven key phenomenological dimensions of their thoughts: (1) attentional engagement, (2) sense of presence, (3) dynamism, (4) involuntary nature, (5) valence, (6) narrativity, and (7) temporality. To analyse these data, we computed—for each participant and separately for each episode type—mean ratings for mind-wandering episodes (characterized by diverse, minimally engaging thoughts) and daydreaming episodes (characterized by coherent, engaging narratives). Because participants contributed multiple observations, these mean values were entered into a series of paired-samples t-tests to compare the phenomenological characteristics of mind-wandering and daydreaming. These analyses were conducted on the subset of participants who reported both episode types (N = 61).

The paired-samples t-tests confirmed significant differences between daydreaming and mind-wandering across most phenomenological dimensions. Daydreaming episodes were more dynamically organized around a single theme, whereas mind-wandering episodes involved shifts across multiple themes. Moreover, daydreaming was rated as more attentively engaging, associated with greater meta-awareness, more narrative in nature, including greater perceived control over the development of thoughts, and accompanied by a stronger sense of presence. In contrast, mind-wandering episodes were rated as more involuntary. No significant difference was observed for valence or attention to the environment. Full results are presented in Table 2.

**Table 2:** Comparison of phenomenological dimensions between daydreaming and mind-wandering (paired-samples t-tests).

Dimension	MW (SD)	DD (SD)	*t*(60)	*p(FDR)*	*Cohen’s d*	95% CI
Attentional engagement (attention on thoughts flow)	3.75 (1.06)	4.92 (0.83)	− 8.55	< .001	1.10	[− 1.44, − 0.89]
Attentional engagement (attention to environment)	5.01 (1.11)	4.84 (0.92)	1.39	.19	0.18	[− 0.08, 0.43]
Attentional engagement (meta-awareness)	4.05 (1.09)	4.64 (0.77)	− 4.47	< .001	0.57	[− 0.86, − 0.33]
Sense of presence	3.50 (1.20)	4.32 (1.09)	− 5.81	< .001	0.74	[− 1.10, − 0.54]
Dynamism	2.51 (0.93)	5.37 (0.96)	− 15.65	< .001	2.00	[− 3.23, − 2.50]
Involuntary nature	0.82 (0.21)	0.65 (0.20)	5.02	< .001	0.64	[0.10, 0.23]
Valence	4.49 (0.85)	4.54 (0.70)	− 0.54	.59	0.07	[− 0.25, 0.15]
Narrativity (self)	3.71 (1.31)	4.25 (1.10)	− 3.89	< .001	0.50	[− 0.82, − 0.26]
Narrativity (others)	3.82 (1.63)	4.23 (1.54)	− 2.36	.03	0.30	[− 0.75, − 0.06]
Narrativity (control)	4.20 (1.26)	4.86 (0.98)	− 3.80	< .001	0.49	[− 1.01, − 0.31]

The table reports results of paired-samples t-tests comparing mean phenomenological ratings for mind-wandering and daydreaming episodes, with FDR-corrected *p*-values.

#### Exploratory analyses: episode-level mixed-effects models

In addition to the preregistered participant-level analyses, we conducted exploratory episode-level mixed-effects models to examine whether the observed differences between mind-wandering and daydreaming were robust at the level of individual episodes (Table 3). For continuous dependent variables, linear mixed-effects models were estimated with Form (MW vs. DD) as a fixed effect and a random intercept for participant. For binary outcomes, generalized linear mixed-effects models with a binomial link function were estimated using the same random-effects structure. This approach allowed us to include all individuals who reported at least one eligible episode (N = 70), unlike the paired-samples t-tests, which required participants to have recorded both mind-wandering and daydreaming episodes.

**Table 3:** Comparison of phenomenological dimensions between daydreaming and mind-wandering (multilevel models).

Dimension	MW (M)	DD (M)	DD—MW (β)	SE	t	*p* (FDR)	ICC	95% CI
Attentional engagement (*attention on thoughts flow*)	3.71 (1.62)	4.92 (1.49)	1.31	.08	16.50	< .001	.21	[1.16, 1.47]
Attentional engagement (*attention to environment*)	5.11 (1.50)	4.85 (1.52)	− .20	.08	− 2.59	.011	.25	[− .35, − .05]
Attentional engagement (meta-awareness)	3.93 (1.70)	4.63 (1.57)	.70	.09	8.08	< .001	.18	[0.53, 0.87]
Sense of presence	3.77 (1.82)	4.38 (1.84)	.83	.09	8.82	< .001	.23	[0.65, 1.02]
Dynamism	2.54 (1.37)	5.53 (1.24)	2.84	.07	42.0	< .001	.21	[2.71, 2.98]
Involuntary nature	.82 (.38)	.63 (.48)	− 1.10	.15	− 7.48	< .001	–	[0.25, 0.45]
Valence	4.48 (1.36)	4.50 (1.62)	.06	.09	.69	.49	.12	[− 0.11, 0.23]
Narrativity (self)	3.83 (2.12)	4.24 (2.23)	.54	.12	4.66	< .001	.19	[0.31, 0.77]
Narrativity (others)	4.03 (2.28)	4.34 (2.35)	.53	.11	4.75	< .001	.35	[0.31, 0.74]
Narrativity (control)	4.15 (1.88)	4.79 (1.74)	.75	.09	7.98	< .001	.21	[0.57, 0.94]

For involuntary nature, results are based on a logistic mixed-effects model; ICC is not applicable.

The table reports results of mixed-effects models with form (mind-wandering vs. daydreaming) as a fixed effect and participant as a random intercept, with FDR-corrected *p*-values.

Across dimensions, the multilevel results closely replicated the paired-samples findings. Relative to mind-wandering episodes, daydreaming episodes were characterized by higher attentional engagement, greater meta-awareness of the ongoing stream of thought, greater sense of presence and higher coherence (i.e., single-topic focus). Daydreaming episodes were also more narrative in nature, involving both self and other characters as well as greater perceived control over the development of thoughts, whereas mind-wandering episodes were more involuntary. Valence did not differ significantly between the two forms. Notably, attention to the external environment—non-significant in the paired t-tests—emerged as a significant predictor in the mixed-effects models. When accounting for within-person variability and differences in the number of recorded episodes, mind-wandering episodes were associated with greater attention to the surrounding environment than daydreaming episodes. This pattern suggests that daydreaming may involve a more actively maintained and volitionally shaped experience.

Intraclass correlations (ICC ≈ 0.12–0.35) indicated modest clustering at the participant level, suggesting that most variability in phenomenological ratings arose from differences between episodes rather than stable differences between individuals. To control for multiple comparisons, the alpha level was corrected using the False Discovery Rate (FDR) procedure^53^. Together, the paired-samples t-tests and multilevel models converged on a consistent phenomenological dissociation between the two forms of cognitive phenomena.

Due to the categorical nature of the temporality variable, episode-level generalized linear mixed-effects models were estimated to examine differences in the temporal structure of mind-wandering and daydreaming. Compared to mind-wandering, daydreaming episodes were significantly less likely to involve multiple temporal elements (OR = 0.31, 95% CI [0.22, 0.43], *p* < 0.001), indicating greater temporal coherence. Daydreaming episodes were also significantly more likely than mind-wandering episodes to include fanciful content (OR = 3.34, 95% CI [1.73, 6.44], *p* < 0.001). A marginal effect suggested that daydreaming episodes may be more likely to involve past-related content (OR = 1.65, *p* = 0.05), whereas no reliable differences emerged for future-oriented episodes indicating that both mind-wandering and daydreaming were similarly likely to involve thoughts about the future. Taken together, these findings suggest that the primary temporal distinction between mind-wandering and daydreaming lies not in their orientation toward specific time frames (past vs future), but rather in the overall temporal coherence and complexity of the experience.

## Discussion

The present study provides the first empirical comparison of mind-wandering and daydreaming as they naturally occur in daily life. Using an experience-sampling design and preregistered analytical procedures, we identified consistent phenomenological differences between the two states across both person-level and episode-level analyses. Participants reported episodes of contemplation multiple times per day and rated them on key phenomenological dimensions, allowing us to capture how mind-wandering and daydreaming are experienced in real time.

Across analytical approaches, daydreaming was characterized by greater thematic coherence, stronger attentional engagement, a richer sense of presence, and more pronounced narrativity, whereas mind-wandering was more like train of thoughts and involuntary in nature. These findings offer empirical support for theoretical accounts proposing that spontaneous thought comprises qualitatively distinct forms rather than a single homogeneous construct. Importantly, the convergence between paired-samples t-tests and mixed-effects models demonstrates that this phenomenological dissociation between mind-wandering and daydreaming is robust at both the participant and episode levels, extending prior laboratory and conceptual work into everyday cognition.

To further elucidate the nature of this dissociation, the sections below examine each of the core phenomenological dimensions—attentional engagement, dynamism, temporality, sense of presence, involuntary nature, and narrativity—and situate the present findings within existing theoretical and clinical models of spontaneous thought.

### Attentional engagement

It is well established that attention plays a central role in the phenomenon of mind-wandering. A widely accepted definition describes mind-wandering as task-unrelated thought, emphasizing a shift of attention from the external environment to internally generated cognitions^2,3^. However, little is known about how attentional dynamics evolve over the course of such episodes. Given that attention—particularly in its extreme form, absorption—plays a crucial role in maladaptive daydreaming, we hypothesized that the degree of attentional engagement may represent a defining feature distinguishing daydreaming from mind-wandering. Recent phenomenological analyses support this idea. Lawson and Thompson^35^, for example, propose immersion as a key criterion differentiating daydreams from other mental states. Our findings align with this proposal: daydreaming was experienced as markedly more attentionally engaging than mind-wandering, with participants reporting a stronger focus on their thoughts and reduced awareness of the surrounding environment. This pattern suggests that attention is less divided between internal and external contexts during daydreaming than during mind-wandering, resulting in a more engrossing, immersive experience. Moreover, daydreaming was associated with greater meta-awareness, indicating that individuals were more likely to recognize that they were currently engaged in an ongoing episode than during mind-wandering. This finding suggests that daydreaming may involve greater engagement of cognitive control processes.

### Dynamism

Researchers have increasingly argued that defining mind-wandering solely in terms of task-unrelatedness is insufficient for distinguishing it from other spontaneous mental states because such definitions do not capture how thoughts evolve over time. Within this broader framework, mind-wandering is typically classified as any thought unrelated to the task at hand^30,31^, but this definition offers limited insight into the structure or dynamics of the thought process itself. The present findings provide evidence for a dynamic distinction between the two states. Mind-wandering was characterized by freely shifting thoughts that moved from one topic to another with relatively limited engagement, as is consistent with descriptions of mind-wandering as a fluid, loosely guided stream of cognition. Daydreaming, in contrast, tended to remain focused on a single coherent theme and was supported by greater attentional involvement. This monothematic and sustained pattern aligns with theoretical accounts conceptualizing daydreaming as a more organized and immersive form of imaginative mentation. Our results therefore empirically support the proposal that mind-wandering and daydreaming differ not only in content or attentional qualities but also in their temporal dynamics.

### Temporal focus

The present findings align with theoretical perspectives arguing that distinctions between different forms of spontaneous thought emerge most clearly when their temporal dynamics are considered. Whereas mind-wandering episodes frequently involved multiple temporal elements within a single episode, consistent with a freely evolving stream of thought, daydreaming episodes were characterized by greater temporal coherence, suggesting a more temporally stable organization of thought.

### Sense of presence

Participants also reported a markedly greater sense of presence during daydreaming compared to mind-wandering. This finding aligns with clinical and theoretical accounts describing daydreaming as an immersive imaginative experience, often accompanied by vivid sensory and emotional realism^42–44,46^. The strong presence dimension observed here supports the idea that daydreaming, even in non-clinical contexts, shares structural features with more immersive forms of imaginative engagement, including maladaptive daydreaming—most notably the feeling of being ‘in’ an imagined scene or narrative. Importantly, however, the present data make clear that such immersion is not inherently maladaptive. Instead, it likely reflects a non-pathological continuum of absorptive imaginative engagement that can occur in daily life without impairing functioning. These findings therefore help clarify that immersive presence is a core experiential marker of daydreaming, even outside clinical presentations.

### Involuntary nature

Our findings also contribute to recent conceptualizations of spontaneous thought, such as Lawson and Thompson’s^35^ proposal that daydreaming constitutes a form of spontaneous immersive imagination. While our data support the idea that immersion reliably differentiates daydreaming from mind-wandering, the spontaneous nature of these states appears more nuanced. Although both phenomena fall under the umbrella of spontaneous thought, participants reported daydreaming as relatively more intentional than mind-wandering. In many cases, individuals described deliberately initiating a daydream, whereas mind-wandering tended to arise more automatically. This pattern suggests a gradient of volitional involvement within spontaneous cognition, with daydreaming occupying a more deliberate position than mind-wandering. This interpretation aligns with prior work showing that mind-wandering can occur both intentionally and unintentionally^9^. It also resonates with clinical descriptions of maladaptive daydreaming as an intentionally initiated and consciously sustained imaginative state^42,54^, which can be viewed as an extreme expression of this volitional engagement. These distinctions have important implications for theories of spontaneous thought: whereas mind-wandering may largely reflect unconstrained or unguided cognitive drift, daydreaming appears to involve a greater degree of voluntary entry and maintenance, even when it occurs outside clinical contexts.

### Narrativity

Daydreaming also exhibited a stronger narrative structure than mind-wandering, consistent with theoretical accounts that describe daydreams as organized, story-like forms of imaginative thought^33^. Participants’ ratings indicated that daydreaming episodes were more likely to involve characters and perspectival structure—features that are far less characteristic of mind-wandering’s more fragmented and loosely connected thought flow. Importantly, during daydreaming episodes, individuals reported greater control over the content of their thoughts, reflecting an increased ability to influence how the episode unfolded, in clear contrast to the unguided, loosely connected train of thought characteristic of mind-wandering.

This narrative quality resonates with clinical observations that, in extreme cases, daydreaming can take the form of maladaptive daydreaming—a highly immersive and elaborated fantasy activity that disrupts daily functioning and can cause significant distress^43,44,46^. While the present study focuses on non-clinical populations, the findings suggest that everyday daydreaming may represent an adaptive analogue of these more elaborated narrative experiences. In this sense, narrativity appears to be a core structural feature of daydreaming, distinguishing it from the more fluid, non-thematic nature of mind-wandering.

Taken together, the present findings have important implications for theoretical accounts of spontaneous thought. Rather than supporting a unitary conception in which mind-wandering and daydreaming differ only in degree, the observed phenomenological dissociations suggest that these states constitute qualitatively distinct modes of spontaneous cognition. The consistent differences in attentional engagement, dynamism, sense of presence, volitional involvement, and narrativity indicate that spontaneous thought is best conceptualized as a heterogeneous class of mental phenomena with partially overlapping but dissociable experiential profiles. This view aligns with recent theoretical proposals emphasizing the multidimensional and dynamic nature of spontaneous cognition, and it extends these frameworks by providing empirical evidence from everyday experience.

More broadly, the present results challenge definitions of mind-wandering that rely primarily on task-unrelatedness and instead highlight the importance of internal structure and experiential dynamics. By demonstrating that spontaneous thought can be both immersive and relatively volitional—as in the case of daydreaming—the findings call for models that accommodate gradations of attentional engagement, narrative organization, and intentional engagement within spontaneous cognition. Distinguishing mind-wandering from daydreaming on phenomenological grounds may therefore help refine existing theoretical taxonomies and improve the precision of experimental paradigms designed to study spontaneous thought in both laboratory and naturalistic settings.

## Limitations

One potential limitation of the present study relates to the procedure used to classify episodes of mind-wandering and daydreaming. In the present study, participants classified their reported episodes according to the defining dimensions of the phenomena introduced during the briefing session—specifically, the degree of attentional engagement and the dynamics of thought. While this procedure allowed us to operationalize and empirically compare daydreaming and mind-wandering, it may have also oriented participants toward these specific dimensions when making their judgments. Several features of the study design, however, help mitigate this concern. Participants were provided with detailed examples of everyday contemplative experiences and received clear explanations of all questions before data collection began. Moreover, the study included follow-up items assessing attentional focus and thematic coherence for each reported episode. The strong correspondence between participants’ categorical classifications and their ratings on these scales—despite participants being unaware of the expected theoretical relationships—suggests that their responses reflected genuine experiential differences rather than simple compliance with predefined criteria. The use of self-categorization was a deliberate methodological decision, consistent with the exploratory aim of delineating phenomenological boundaries between two closely related forms of spontaneous thought. Future research could build on this work by examining these distinctions without explicitly introducing attentional engagement and thought dynamics as defining features. Such studies would help determine whether similar patterns emerge when participants classify their experiences more intuitively and would further clarify the structure of spontaneous thought in everyday life.

## Final conclusions

The widespread interchangeability of the terms mind-wandering and daydreaming has contributed to conceptual ambiguity in the literature, with potential consequences for theory, measurement, and methodology. Addressing this issue requires systematic investigation of the ways in which these phenomena resemble or differ from one another in everyday cognition. The present study sought to clarify these distinctions by examining the phenomenological characteristics and content of mind-wandering and daydreaming as they naturally unfold in daily life.

Using an experience-sampling method, we captured real-time reports of both states and identified robust differences across multiple dimensions, including attentional engagement, thematic coherence, sense of presence, and narrativity. To our knowledge, no prior study has empirically verified such distinctions in real-world experience. Together, these findings provide supporting evidence that mind-wandering and daydreaming represent qualitatively distinct forms of spontaneous cognition rather than interchangeable labels for the same cognitive phenomenon. Importantly, our data show that both mind-wandering and daydreaming can occur during task engagement as well as during task-unrelated moments. Given that mind-wandering is often defined as the presence of thoughts unrelated to the current task, this observation highlights the need to reconsider a strict association between mind-wandering and task disengagement. Mind-wandering may therefore arise even in the absence of an explicit task, reflecting a mode of spontaneous cognition that occupies the mind over time rather than merely a withdrawal from ongoing activity—an interpretation that warrants further investigation in future research.

## Data Availability

The data that support the findings of this study are publicly available at: 10.57903/UJ/Q0JSPH.

## Appendix

Design table.

**Table:**

Question	Hypothesis (if applicable)	Sampling plan (e.g. power analysis)	Analysis plan	Interpretation given to different outcomes
How mind wandering and daydreaming differ in terms of cognitive and phenomenological features such as attentional engagement, dynamism, sense of presence, involuntary nature, narrativity, valence, temporality and task-unrelatedness?	Elucidating differences between mind-wandering and daydreaming: Mind-wandering, compared to daydreaming, is characterized by higher ratings of freely moving from thought to thought and lower ratings of attentional engagement, sense of presence, and narrativity. Daydreaming should exhibit higher ratings for attentional engagement, sense of presence, narrativity, and a dynamic tendency to remain fixed on a single theme. As to intentionality, valence and temporality there are no firm predictions, and for these features, the present project will be exploratory in nature	No data are available regarding the effect sizes of differences between mind-wandering and daydreaming. Therefore, the analysis is based on typical effect sizes (*Cohen’s d*) considered small, medium, and high: 0.2 (η^2^ equivalent 0.01), 0.5 (η^2^ equivalent 0.06), and 0.8 (η^2^ equivalent 0.14), respectively^48,49^. The required sample sizes for 80% power in the two-tailed test are 199, 34, and 15, respectively. A sample size of about 70 participants was assumed sufficient to detect a medium effect. Sensitivity analysis suggests that this sample size enables the detection of an effect size of 0.34 (η^2^ equivalent 0.03; which may generally be considered closer to a small effect size)	For each participant, and separately for each type of instance of mind-wandering (thinking about diverse, unrelated subjects with minimal engagement) and daydreaming (focusing on a singular topic, with thoughts forming a coherent narrative and sustained engagement), we will calculate mean ratings (e.g., mind-wandering dynamism rating; daydreaming dynamism rating) for all dependent variables because subjects will provide dependent multiple observations. Accordingly, a dependent t-test will be conducted to find differences between the phenomenological characteristics of mind-wandering and daydreaming. To control for multiple comparisons, we will correct the alpha level using the False Discovery Rate correction^53^	While we expect daydreaming and mind-wandering to differ from each other in the discussed terms, an alternative possibility is that they will not differ, suggesting that participants treat them as the same constructs. Such a result, due to the lack of control over the retrieval phase in an experience sampling method, would necessitate future laboratory-based studies to ensure strict control over the retrieval phase and participants’ responses to questions. This would help limit the risk of retrospective biases when answering questions about phenomenological characteristics

## Code availability

Statistical analyses were conducted in R (version 4.4.2). The analysis scripts used to generate the reported results are available from the corresponding author upon reasonable request.
